# Cooperative Position Aware Mobility Pattern of AUVs for Avoiding Void Zones in Underwater WSNs

**DOI:** 10.3390/s17030580

**Published:** 2017-03-13

**Authors:** Nadeem Javaid, Mudassir Ejaz, Wadood Abdul, Atif Alamri, Ahmad Almogren, Iftikhar Azim Niaz, Nadra Guizani

**Affiliations:** 1 COMSATS Institute of Information Technology, Islamabad 44000, Pakistan; mudassirejaz1@gmail.com (M.E.); ianiaz@comsats.edu.pk (I.A.N.); 2 Research Chair of Pervasive and Mobile Computing, College of Computer and Information Sciences, King Saud University, Riyadh 11633, Saudi Arabia; aabdulwaheed@ksu.edu.sa (W.A.); atif@ksu.edu.sa (A.A.); ahalmogren@ksu.edu.sa (A.A.); 3 Department of Electrical and Computer Engineering, Purdue University, West Lafayette, IN 47907, USA; nguizani@purdue.edu

**Keywords:** acoustic communication, underwater wireless sensor networks, autonomous underwater vehicle, underwater glider, cooperative routing, void zone

## Abstract

In this paper, we propose two schemes; position-aware mobility pattern (PAMP) and cooperative PAMP (Co PAMP). The first one is an optimization scheme that avoids void hole occurrence and minimizes the uncertainty in the position estimation of glider’s. The second one is a cooperative routing scheme that reduces the packet drop ratio by using the relay cooperation. Both techniques use gliders that stay at sojourn positions for a predefined time, at sojourn position self-confidence (s-confidence) and neighbor-confidence (n-confidence) regions that are estimated for balanced energy consumption. The transmission power of a glider is adjusted according to those confidence regions. Simulation results show that our proposed schemes outperform the compared existing one in terms of packet delivery ratio, void zones and energy consumption.

## 1. Introduction

Underwater wireless sensor networks (UWSNs) consist of sensor nodes and sinks or vehicles that cooperate to collect data from the aquatic environment. They have been deployed to perform monitoring tasks like oceanographic data collection, military surveillance, pollution monitoring, natural disaster prevention, navigation, etc. [1]. However, due to dynamic nature of the aquatic environment, it faces many challenges, like the propagation speed of the acoustic signal in water that results in large propagation delay, the available bandwidth of the acoustic channel being limited and depending on the transmission range and the frequency, temporary loss of connectivity, high BER, etc. [2].

Therefore, autonomous underwater vehicles (AUVs) are widely used for oceanography in the aquatic environment to collect data from sensor nodes, due to their capability to monitor an environment that is difficult to reach. For example, AUVs are used by the oil and gas industry to make maps for seafloor before building underwater infrastructure: pipelines can be installed with minimum cost in an effective manner. AUVs also allow survey companies to conduct precise surveys with low cost as compared to traditional survey methods. They are also used for surveillance, anti-submarine warfare, information operations, etc. [3].

To achieve high network throughput, cooperative routing is used by utilizing the fact that sensor nodes of the network can overhear transmission from each other, in which relay nodes are selected in a priority order to transmit their data packets, if a top priority node fails to deliver a data packet within the predefined signal to noise ratio (SNR) [4,5,6]. Then, two or more copies of the same data packet are transmitted to the destination via relay nodes. The cooperative routing is used to minimize the packet loss by combining packets at the destination via diversity techniques. However, this consumes more energy and increases end to end delay [2].

To increase the end to end reliability and avoid void zones, position information is very important because void zones (area or volume remains un-sensed or unvisited by the mobile sinks or AUVs during the underwater communication) increase data loss that results in a short network lifespan. Although AUVs visit the network periodically (AUVs change their location after defined interval) by using global positioning system (GPS) to locate themselves, however, due to the dynamic nature of the underwater environment, it faces localization errors, drifting due to water currents, etc., resulting in position uncertainty of AUVs. Such uncertainties decrease the reliability, efficiency and quality of the collected data [1].

AUVs (gliders) are used to minimize uncertainties by using predictable trajectories (sawtooth trajectories) that are used to predict positions. By using those predictable trajectories, energy consumption can be minimized; however, if the mobility pattern is predefined, then void zones increase with the increase in the sparsity of the network field [1], resulting in less network throughput. Therefore, in this paper (an extension of [7]), two schemes are proposed: the first one is the position-aware mobility pattern (PAMP) to avoid void zone creation, and the second one is the cooperative PAMP (Co PAMP) that achieves a high packet delivery ratio. PAMP avoids the void zone by setting the dynamic mobility pattern of gliders with predictable trajectories. It computes self-confidence (s-confidence) and neighbor-confidence (n-confidence) regions to pick sojourn positions in order to minimize the distance between the source and the destination. Similarly, Co PAMP selects two best relay gliders (that received data packets successfully from the source glider) to perform cooperative routing to receive data within the predefined SNR threshold at the destination. The simulation results show that our recent proposed schemes outperform the compared baseline existing scheme in terms of packet delivery ratio, energy consumption and avoiding void zones.

The rest of the paper is organized as follows. In Section 2, related work is presented, and our proposed schemes are described in Section 3. Simulation results are discussed in Section 4. Finally, Section 5 concludes our proposed work.

## 2. Related Work

Many routing protocols have been proposed to avoid void zones, achieve high network throughput and minimize energy consumption. Existing routing protocols about position uncertainty, mobility and cooperative routing are discussed in this section.

### 2.1. Protocols for Position Uncertainty

In [1], the authors propose an optimization technique based on the position estimation and uncertainty regions around the gliders. Two types of uncertainty regions are discussed in this technique: internal-uncertainty region and external-uncertainty region. The source glider specified the internal-uncertainty region, whereas the external-uncertainty region is defined by the neighboring gliders. The trajectory of the gliders is predefined (sawtooth trajectory), and they adjust their transmission power according to these uncertainties of regions.

With the help of light beacons and a camera, the position of the AUV is estimated in [8]. The set of light beacons or light markers is placed on the AUV and detected using camera. After detecting light markers, the 3D position of the AUV is estimated; however, this tracking system is for very short range, that is up to 10 m.

In [9], an AUV positioning algorithm is proposed based on the strap down inertial navigation system (SINS) and the long baseline (LBL) acoustic positioning system. The seabed hydrophones are used by the underwater LBL to confirm the position of the AUV; at least three hydrophones are attached at the bottom of the mother ship, and those receive signals from the hydrophone placed at the seabed. Using the LBL positioning system, the 3D position of each hydrophone is calculated, which is then used by AUVs for local area positioning.

The authors propose a novel scheme for estimation and navigation of the AUV state in the presence of water currents [10]. They used the extended Kalman filter (EKF) to estimate the state of AUV and water currents in the north and the east directions. This estimation process is integrated with the speed through water (STW) to give a better estimation of the speed over ground (SOG). Using this information, the navigation and the AUV state estimation are performed.

A paradigm-changing geographic routing protocol is presented in [11], to model the position uncertainty, and this approach relies on the statistical approach. The simulation results are validated by using real-time test bed emulations. These emulations use underwater acoustic modems. The performance tradeoffs of position-based schemes are given in Table 1.

In [1,8,9,10,11], the energy consumption and packet drop ratio is high. In order to reduce the energy consumption and increase the packet delivery ratio, we have proposed two schemes, PAMP and Co PAMP. PAMP minimizes the energy consumption and end to end delay; whereas, Co PAMP improves the packet delivery ratio by avoiding the void regions.

### 2.2. Mobility Focused Protocols

The authors in [12] use AUVs and gateway nodes to maximize the data delivery ratio and to reduce the energy consumption. In the AUV-aided underwater routing protocol (AURP), distant data transmissions are minimized by using AUVs as relay nodes. Normal nodes send their sensed data to the gateway nodes, then AUVs collect those data and forward them to the sink. The three-dimensional sink mobility (3D-SM) scheme is proposed in [13], in which the authors divide the three-dimensional network volume into four rectangular cuboids (RC). In one cuboid, a mobile sink (MS) is deployed to gather data packets from sensor nodes, and in the remaining three cuboids, courier nodes (CNs) are deployed to gather data from nodes at relatively shorter distances. The energy consumption of normal nodes is minimized due to MS and CNs due to shorter distances between the source node and the destination (MS or CN).

A network of randomly-deployed identical sensor nodes is considered in [14]. In this scheme, the sensed data are gathered from pathway nodes, and the AUV visits pathway nodes to save energy by avoiding distant transmissions. An efficient data-gathering (AEDG) routing protocol is proposed in [15]. Sensor nodes in the network are associated with gateway nodes, and gateway nodes gather the data from sensor nodes. An AUV visits gateway nodes to collect the data and ensures the reliability. To avoid gateway nodes being overloaded, the authors limit the number of sensor nodes associated with the gateway nodes.

In [16], a routing protocol is proposed in which three regions or potential fields are defined, and the network area is divided into three different layers based on: the depth, residual energy and density of the sensor nodes. The authors in [17] propose a link state-based adaptive feedback routing (LAFR) protocol for underwater acoustic sensor networks (UASNs). By using the link state detection mechanism, the link state is determined, then LAFR uses the link state information in the adaptive feedback method to fully utilize the asymmetric links underwater for conserving energy and to maintain routing tables.

The channel-aware routing protocol (CARP) [18] uses link quality information for data forwarding. For the selection of relay nodes, the authors propose that the nodes are selected as relay nodes, if they exhibit the latest history of successful data transmissions. By using hop count, CARP avoids loops to successfully route around void and shadow zones. In [19], a void-aware pressure routing (VAPR) protocol is proposed that uses enhanced beacons and opportunistic directional data forwarding in the network to propagate control information to sensor nodes. Sensor’s depth, hop count to the sonobuoy and the data forwarding direction of a sensor node are included in the control information.

The coverage hole problem is addressed in [20]. Taking the benefit of the redundant overlapping of the ranges of sensor nodes, the coverage hole is repaired during network operation. This protocol reduces the energy consumption, resulting in a high network throughput and lifetime of the network at the cost of high end to end delay. The performance tradeoffs of mobility-based schemes are given in Table 2.

The discussed existing schemes [13,14,15,16,17,18,19] have limitations in terms of high end to end delay. Therefore, in this paper, PAMP is proposed to minimize the end to end delay.

### 2.3. Cooperative Routing Protocols

Cooperative routing takes the benefit of the broadcast nature of wireless communication in which the transmitted signal is overheard by unintended sensor nodes within the transmission range. It is an alternate to multi-hop communication for reducing link impairments and fading. In [21], the authors propose an opportunistic routing protocol for UWSNs. They used the local topology information and adopted a greedy method for finding the set of nodes that made maximum progress with limited hidden terminals. In order to minimize hidden terminals, relay nodes are selected as the destination.

The authors propose in [22] a mathematical model for cooperative routing in UWSNs to reduce the end to end delay by calculating the dominating set (DS) of a given network as a digraph. The DS formation allows the optimal formation of sensor nodes for cooperative routing; it reduces the communication overhead and minimizes the energy consumption of the network. In [23], the authors propose the cross-layer minimum collision cooperative routing (MCCR) algorithm for cooperative transmission and power allocation to overcome collision probability.

Tan et al. [24] propose a cooperative transmission routing protocol for UWSNs. The forwarder nodes are selected on the bases of distance cost and local measurement of the channel information. This routing protocol considers cooperative transmission at the routing layer, as well as cooperative transmission at the physical layer. In [25], the authors propose a cooperative routing protocol in which it is first determined whether cooperative routing is required or not. If required, an optimal relay is selected, which fulfills the SNR constraints. In addition, the Bellman–Ford routing technique along with mixed integer linear programming (MILP) are also used to achieve the minimum energy consumption during cooperative routing.

In [26], the authors propose a collision minimization strategy using cooperative routing. They formulated the problem using mixed integer non-linear programming (MINLP). In this scheme, MINLP problems are solved by minimizing the search space with branch and bound algorithm. The authors propose [27] to achieve energy efficiency by allocating power at each hop from the source to the destination. Each node calculates its cost function based on the residual energy, distance and SNR of the link. This routing protocol is efficient in terms of end to end delay and network lifetime. The performance tradeoffs of cooperation-based schemes are given in Table 3.

The discussed cooperative routing protocols [21,22,23,24,26,27] have a high ratio of void zones, resulting in a high packet drop ratio. In order to avoid void zones and to enhance the network throughput, Co PAMP is proposed in this paper, which maximizes the packet delivery ratio and avoids the void regions. It is discussed in Section 3.

## 3. Proposed Schemes

In this section, we present PAMP (position-aware mobility pattern) and Co PAMP (cooperative PAMP) for UWSNs. PAMP is proposed to minimize energy consumption and avoid void zone occurrence. Similarly, Co PAMP is presented to enhance the network throughput and to avoid void hole creation using cooperative routing. Details of both schemes are given in this section.

### 3.1. Position-Aware Mobility Pattern

Let there be *n* gliders in the network volume where *N* nodes are deployed. With the help of a pump and magnetic compass, gliders can change their vertical and horizontal motion and direction. The concept of sojourn positions [2] is used to minimize the uncertainty in the estimation of the self-confidence (s-confidence) and neighbor-confidence (n-confidence) region. The gliders transmit and receive information during their stay at sojourn positions. The control information, like s-confidence region, direction and present position of a glider, is continuously shared with neighboring gliders during the stay time. On the basis of this information, a glider estimates its future position and the n-confidence region of a neighboring glider.

**Figure 1:**
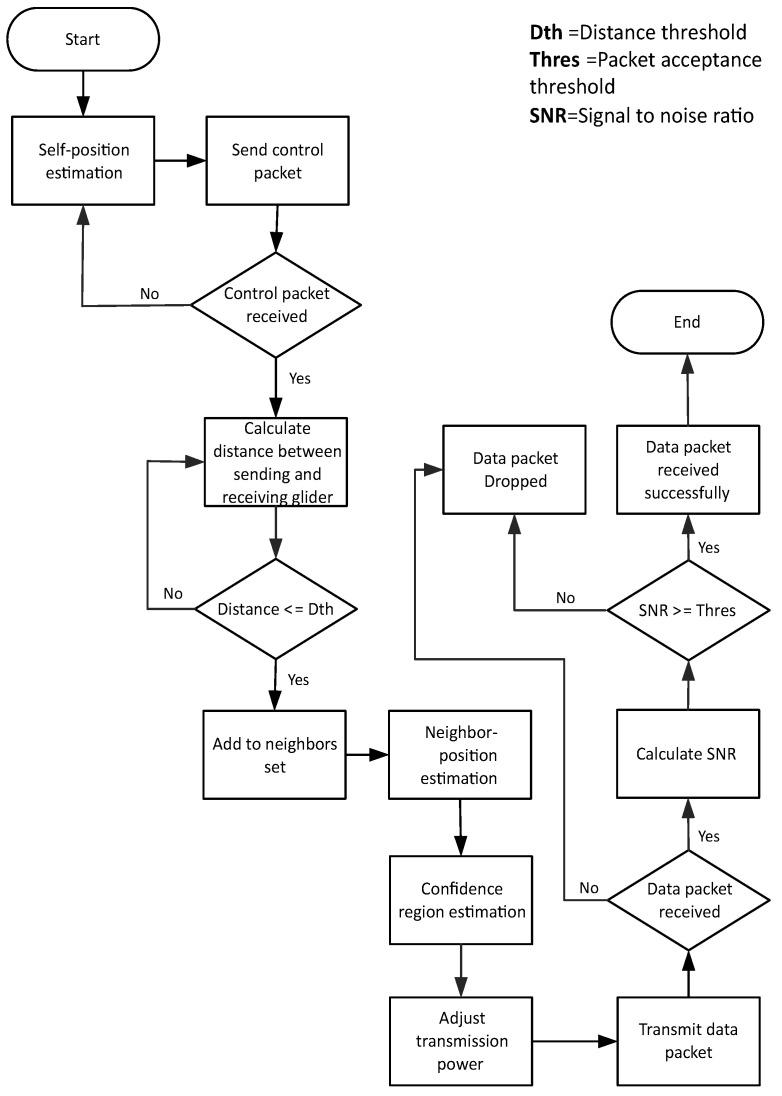
Protocol operation of position-aware mobility pattern (PAMP).

With the help of a magnetic compass, the direction of gliders is maintained, and gliders in the excess zone move outwards in order to avoid the void zone. After the movement, gliders stop at sojourn positions for a predefined ‘stay time’. During this time, control information and data packets are shared between neighbors. On the basis of control information, a transmitting glider (sender) predicts the position of the receiving glider (receiver) and estimates the n-confidence region. On the basis of these estimations, transmission power is adjusted. Uncertainty in the estimation of the position and n-confidence region of any glider may lead to packet drop and the waste of energy in terms of the transmission power. The protocol operation of PAMP is shown in Figure 1.

#### Position Extrapolation

**Figure 2:**
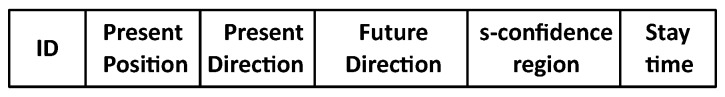
Control information packet format.

The control information shown in Figure 2, which is shared between neighbors, includes present position, future direction and the s-confidence region of gliders.

Depending on the present state and properties, each glider estimates a few parameters, and the s-confidence region is one of these. Basically, volume around the glider ‘seen by a glider itself’ is known as the s-confidence region. At the s-confidence region, the glider is confident that it will successfully receive any packet. The s-confidence region is calculated as follows:

(1) $$SC_{region}=\left(\left(X^{2}+Y^{2}+Z^{2}\right)\leq R^{2}\right)+error\left(p\right)$$

where *X*, *Y* and *Z* are the coordinates of the neighbors in the s-confidence region of the glider. *X*, *Y* and *Z* are used to check whether they lie in the depth range (*R*) of a glider; if yes, they are part of the s-confidence region that is the volume seen by the glider. error(p) is a constant for the uncertainty in the position of a glider, and it is same for all coordinates. The *X*, *Y* and *Z* coordinates are computed as given in Equation (2).

(2) $$\begin{array}{c} X=x_{0}+R\cos\left(L+\frac{\pi }{n}\right)+error\left(p\right)\\ Y=y_{0}+R\sin\left(L+\frac{\pi }{n}+error\left(p\right)\right)\\ Z=z_{0}+R\sin\left(L+\frac{\pi }{n}+error\left(p\right)\right) \end{array}$$

where $x_{0}$, $y_{0}$ and $y_{0}$ are considered as the origin of the glider and *L* is the length of the slope to adjust the angle ($\frac{\pi }{n}$) for the future glider position to adjust the transmission power; which is calculated as given in Equation (3).

(3) $$TP_{total}=\left(\Delta R\right)P_{n+1}+error\left(p\right)$$

$TP_{total}$ represents the total transmission power; (ΔR) is a range for which the transmission power $\left(P_{n+1}\right)$ needs to be adjusted. Therefore, each glider shares its information regarding the s-confidence region with its neighbors to adjust their transmission power.

Suppose that Glider 1 sends a control packet to Glider 2. After the successful reception of the control information, the neighboring glider does not rely only on the s-confidence region; rather, it estimates n-confidence region, which is the region around the glider ‘seen by its neighbors’, and the neighboring glider (Glider 2) is confident that the receiving glider (Glider 1) will successfully receive any packet that reaches this region. It is (n-confidence region) calculated as given in Equation (1). Therefore, it is assumed that transmission power is adjusted on the basis of the n-confidence region, which is (power adjustment) computed according to Equation (3).

Estimation of the confidence regions is performed in order to adjust the transmission power precisely and to minimize the packet error rate $\left(PER={\left(1-BER\right)}^{n}\right)$, which results in a high packet drop ratio. *n* denotes the number of bits transmitted in a packet. The BER is calculated according to Equation (4).

(4) $$BER=\frac{1}{2}erfc\left(\sqrt{S}NR\right)$$

where erfc() denotes the complementary error function, and the SNR is computed by Equation (5).

(5) $$SNR=\frac{\Delta PA(\Delta R,f)}{N(f)\Delta f}$$

ΔP denotes the adjustment made in transmission power $\left(P_{n+1}\right)$ according to the distance between the source and the destination; A(ΔR,f) represents the attenuation on the acoustic channel over a range ΔR (ΔR=(R-r)) for a signal of frequency *f*; and the total attenuation of the transmitted signal is computed on the basis of spreading factor [28] and Thorp model [29] given in Equation (6).

(6) $$10log\left(\alpha \left(f\right)\right)=\left\{\begin{array}{cc} \frac{0.11f^{2}}{\left(1+f^{2}\right)}+\frac{44f^{2}}{\left(4100+f\right)}+0.000275f^{2},\\ & f\geq 0.4\\ 0.002+0.00\left(\frac{f}{\left(1+f\right)}\right)+0.011f,\\ & f\leq 0.4 \end{array}\right.$$

where *α* is absorption loss in dB/km and *f* denotes operating frequency in Hz. Absorption loss α(f) is calculated according to Equation (7).

(7) $$\alpha =\frac{10^{\alpha (f)}}{10}$$

If α(l,f) is the total attenuation of the transmitted signal over path *l* due to spreading loss and absorption loss, then:

(8) 10log(A(l,f))=k10log(l)+l10log(α(f))

where *k* is the spreading coefficient. N(f) is the ambient noise computed by considering four noise components (turbulent $N_{t}$, wind $N_{w}$, shipping $N_{s}$ and thermal $N_{th}$), as given in Equation (9).

(9) $$N\left(f\right)=N_{t}\left(f\right)+N_{w}\left(f\right)+N_{s}\left(f\right)+N_{th}\left(f\right)$$

where:

(10) $$10log(N_{t}\left(f\right))=17-30log\left(f\right)$$

(11) $$10log\left(N_{w}\left(f\right)\right)=50+7.5w^{1/2}+20log\left(f\right)$$

(12) $$10log(N_{s}\left(f\right))=40+20\left(s-0.5\right)+26log\left(f\right)-60log\left(f+0.03\right)$$

(13) $$10log(N_{th}\left(f\right))=-15+20log\left(f\right)$$

The s-confidence region estimation solely depends on the present state of a glider irrespective of the channel state and the distance between neighbors; whereas, underwater, the channel state has a major impact on ongoing communication. Sojourn positions in the network decrease the probability of uncertainty in the estimation of the n-confidence region; however, the volume of a region will always be less than an s-confidence region. The neighboring glider estimates the confidence region by considering the link quality (lq between the source and the destination) and the distance between the receiver and the transmitter. Due to this reason, the n-confidence region is less than the s-confidence region, and the transmission power is adjusted according to the n-confidence region; where lq [30] is computed as given in Equation (14).

(14) $$lq_{d}^{s}=\beta S_{d}^{s}+\left(1-\beta \right)lq_{d}^{s-1}$$

where *β* denotes the confidence factor of receiving the data successfully and $S_{d}^{s}$ denotes the success ratio between the sender and the receiver that depends on the $lq_{d}^{s-1}$ success ratio of the transmissions between the source and the destination after t−1 transmissions.

**Figure 3:**
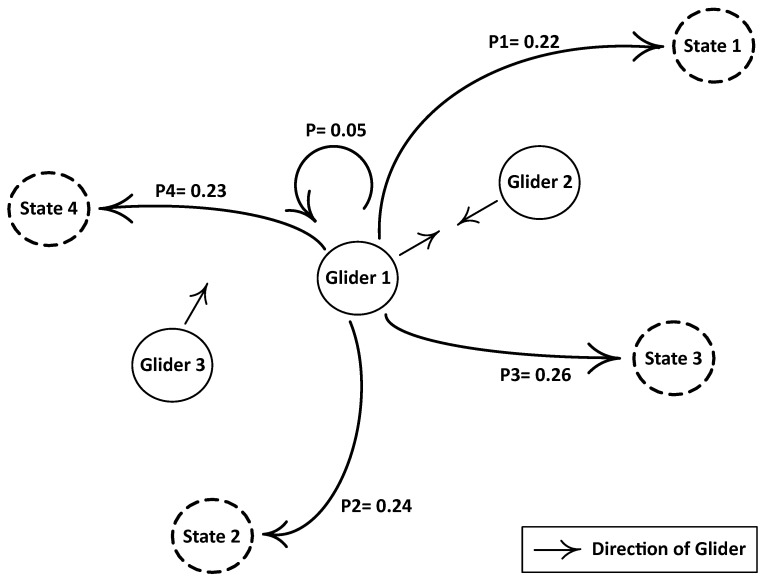
Neighboring gliders are too close.

**Figure 4:**
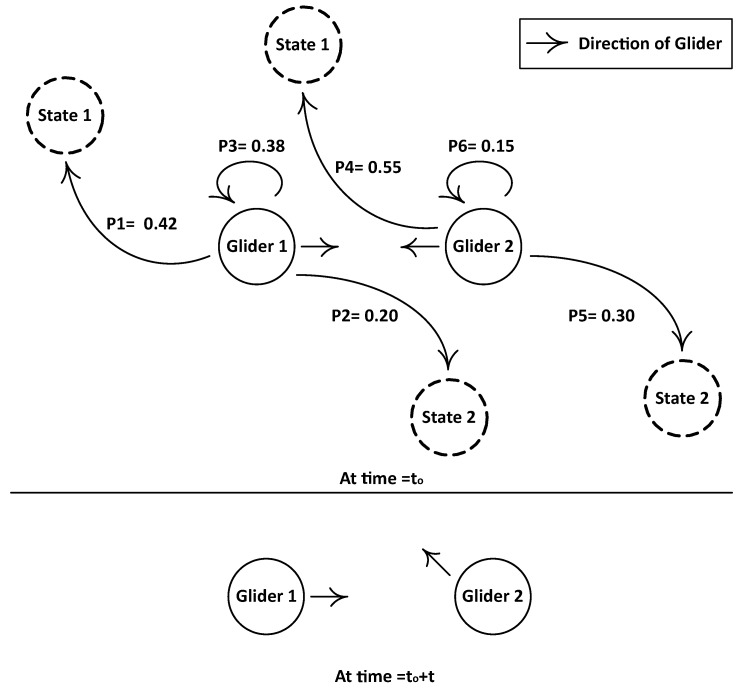
Gliders heading towards each other.

**Figure 5:**
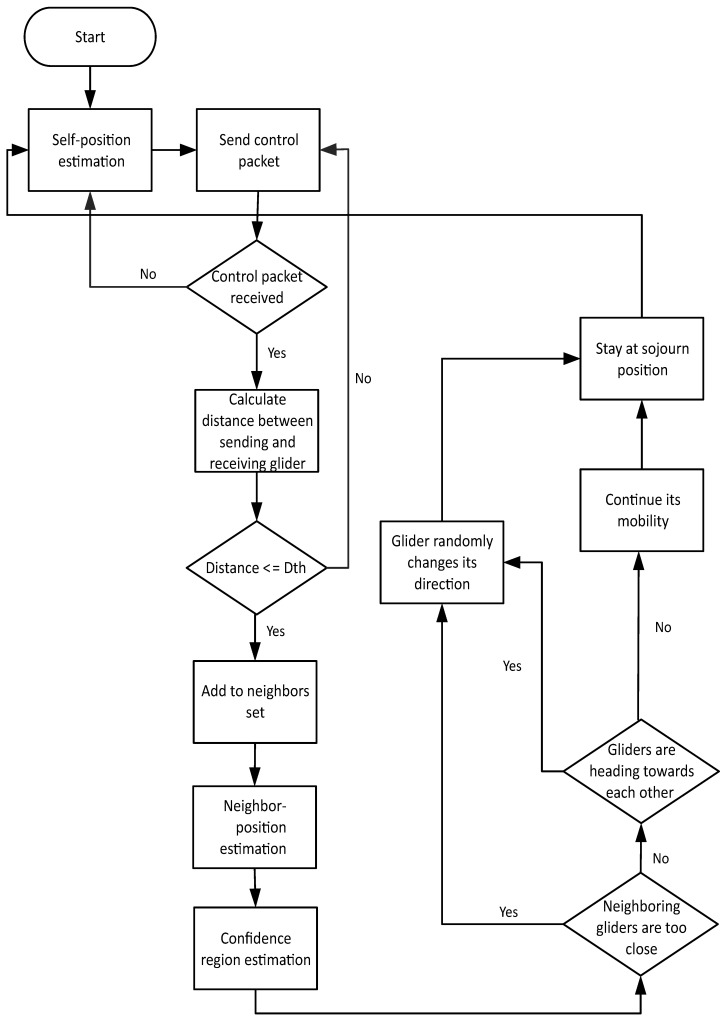
Mobility of gliders in PAMP and Co PAMP.

When gliders move to their next respective locations, the following possibilities may occur:

1. Neighboring gliders are too close
2. Gliders head towards each other
  - Neighboring gliders are too close: Figure 3 shows that Gliders 1, 2 and 3 are too close to each other; as a result, they create an excess zone. Basically, the ‘excess zone’ is an area of the network in which an extra number of sensing nodes or gliders is present as compared to the appropriate number of nodes to fully cover that area.
    Let us suppose that Gliders 1, 2 and 3 are at sojourn positions. Due to the exchange of the control packet, they came to know that they are in the excess zone; then, a glider is randomly selected from the excess zone, and it estimates its future position using the Markov process on the basis of control information shared by its neighbors. Before the lapse of the ‘stay time’ (ST), the predicted direction of a selected glider is shared with the neighbors. The ST is calculated as given below:
    (15) $$ST_{total}=D(s,d)\times T_{rec}$$
    where D(s,d) is the distance between the sending and the receiving glider and $T_{rec}$ shows the time required for receiving data over D(s,d). $T_{rec}$ is computed according to Equation (16).
    (16) $$T_{rec}=T_{rec}\times \left(N_{g}-1\right)$$
    $N_{g}$ denotes the number of gliders involved in data communication, and one is subtracted because the destination will be excluded and rest of the gliders take part in the data transmission.
  - Gliders heading towards each other: Consider Figure 4 for Case 2. Two gliders are at sojourn positions and waiting for a stay time. The control information at time to is exchanged between these two gliders, and the position estimation process takes place. As both gliders are heading towards each other, so, at time to+t, they will be overlapping each others’ course. In order to avoid this situation, one of the gliders needs to change its course. For this reason, the Markov process is performed especially considering the direction of the neighboring glider; then the future direction of the glider for time to+t is shared with the neighbors. The mobility operation of gliders in PAMP is shown in Figure 5.

### 3.2. Cooperative Position-Aware Mobility Pattern

The objective of Co PAMP is to achieve high network throughput. In Co PAMP, two best neighboring gliders of the source glider are selected as the relay, and they cooperate with the source glider to enhance the SNR of the received signal by re-transmitting the data packet at the destination and combine by using the MRC as the diversity technique.

During the data transmission phase, the source glider transmits a data packet towards the destination glider, and neighboring gliders also receive that data packet. When the packet is received at the destination glider, the instantaneous SNR is calculated at the destination as follows [31]:

(17) $$\gamma_{AF}=\gamma_{sg,rg,dg}+\gamma_{sg,dg}$$

where $\gamma_{sg,dg}$ is the link SNR between the source glider and the destination glider and $\gamma_{sg,rg,dg}$ is the equivalent SNR between the source glider and the destination glider through a relay glider. The equivalent SNR of the relayed signal by the glider is calculated as follows [31]:

(18) $$\gamma_{sg,rg,dg}=\frac{\gamma_{sg,rg}\gamma_{rg,dg}}{\gamma_{sg,rg}+\gamma_{rg,dg}+1}$$

If the SNR is greater than the packet acceptance threshold $\left(PA_{th}\geq 0.8\right)$, then the packet is marked as successfully received, and no glider is bothered to relay the same data packet for cooperation towards the destination. On the other hand, if the SNR of the received packet is less than the $PA_{th}$, destination gliders send the negative acknowledgment (NACK) to the source glider. On receiving NACK, the first glider in priority according to the gliding depth relays the data to cooperate with the source glider by sending the same data packet towards the destination. Again, the SNR is checked at the destination; if the SNR is still less than the $PA_{th}$, then the second best relay glider takes part in cooperative communication and transmits the data packet towards the destination. The relationship between the transmitted signal to the source node and relay nodes is computed according to Equations (19) and (20) [31].

(19) $$Y_{\left(sg,rg\right)}=X_{sg}g_{\left(sr,rg\right)}+n_{\left(sg,rg\right)}$$

(20) $$Y_{\left(sg,dg\right)}=X_{\left(sg\right)}g_{\left(sg,dg\right)}+n_{\left(sg,dg\right)}$$

The relayed signal from the relay to the destination is represented as [31]:

(21) $$Y_{\left(rg,dg\right)}=Y_{\left(sg,rg\right)}g_{\left(rg,dg\right)}+n_{\left(rg,dg\right)}$$

$X_{sg}$ is the signal sensed and transmitted by the source glider sg; $Y_{\left(sg,dg\right)}$ and $Y_{\left(sg,rg\right)}$ are the signals received by the destination glider dg and the relay glider rg, respectively. $Y_{\left(rg,dg\right)}$ is the relayed signal from the relay glider rg to the destination glider *d*. $g_{\left(sg,dg\right)}$ is the channel gain from the source glider to the destination glider; $g_{\left(sg,rg\right)}$ denotes the channel gain from the source to the relay glider; $g_{\left(rg,dg\right)}$ represents the channel gain from the relay to the destination glider. $n_{\left(sg,dg\right)}$ shows the channel noise from the source to the destination glider; $n_{\left(sg,rg\right)}$ is the channel noise from the source to the relay glider; and $n_{\left(rg,dg\right)}$ is the channel noise between the relay and the destination glider.

The two signals received at the destination are then combined by using MRC as the diversity combining technique to achieve a better good-put result. We target delay-sensitive applications and considered the amplify and forward mechanism instead of decode and forward, because we aim to achieve high reliability by choosing a route with high lq.

**Figure 6:**
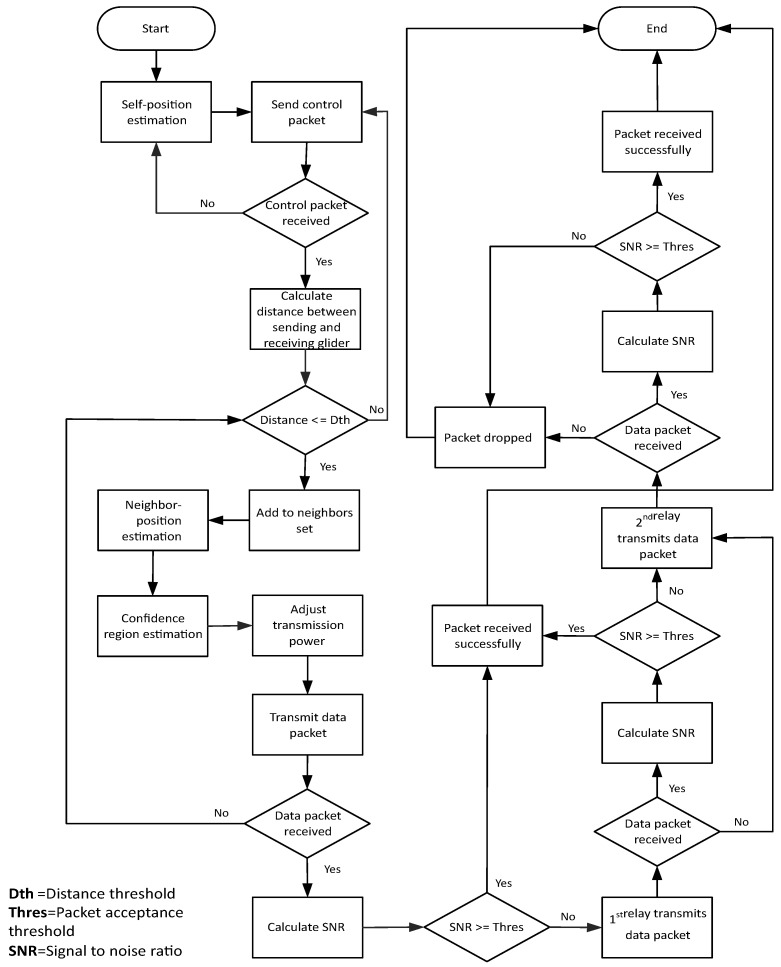
Protocol operation of Co PAMP.

Consider Figure 6 for the protocol operation of Co PAMP. Except cooperative routing, the protocol operation of Co PAMP is the same as that of PAMP.

### 3.3. Throughput Maximization

Linear programming is one of the most widely-used techniques for achieving a best possible outcome of an objective function. It is a special case of mathematical programming in order to optimize the linear function. Few linear constraints and some inequality constraints are defined; those must validate the objective function by giving positive results. We have formulated an objective function to show that the throughput is optimized via reducing the energy consumption by the linear model.

The operations of PAMP and Co PAMP consume energy during the data transmission and the data reception. Therefore, it is necessary that maximum and minimum energy consumption required for data gathering must be known to evaluate our objective function, which is given in Equation (22).

(22) $$Max\sum_{gliders=0}^{gliders_{max}}Th\left(gliders\right)\;\forall \;gliders\in N$$

where gliders are used to sense and collect data packets from the network field and Th shows throughput that is defined as the amount of total data packets received successfully at the destination. Th is presented in Equation (23).

(23) $$Th=\sum_{p=0}^{p_{max}}lq\left(p_{n}\right)\;\forall \;p\in Th$$

$p_{n}$ denotes the total number of packets, and lq represents that only those data packets are considered to satisfy the $PA_{th}$; where lq is given in Equation (24).

(24) $$\begin{array}{c} lq=\left\{\begin{array}{cc} 1 & \mathrm{if}\mathrm{lq}\geq \mathrm{th}\\ 0 & \mathrm{if}\mathrm{lq}<\mathrm{th} \end{array}\right. \end{array}$$

Equation (24) shows that when lq is greater than the threshold th (lq = 1), only then it is counted in network Th. Linear constraints for the objective function are given as follows:

(25a) $$\begin{array}{c} E_{tx,rx}\leq E_{initial}^{max}\;\forall \;i\in N \end{array}$$

(25b) $$\begin{array}{c} E_{\left(required\right)}\geq E_{\left(i\right)}^{min}\;\forall \;i\in N \end{array}$$

(25c) $$\begin{array}{c} lq\geq th\;\forall \;lq,th\in N \end{array}$$

(25d) $$\begin{array}{c} depth_{gliding}\leq depth_{gliding}^{max}\;\forall \;s,d\in N \end{array}$$

Equation (25a) ensures that the energy required for data communication should not exceed the initial energy of a sensor node. Moreover, Equation (25b) shows that the energy needed for gathering data packets must be above the minimum required energy. The lq is checked at every destination glider in order to gather data packets with high SNR. A data packet is counted in throughput only if it has lq = 1, which means it has lq above or equal to the predefined threshold; otherwise, lq = 0 means the packet will not be accounted, and a relay glider will retransmit the data packet to improve the SNR at the destination (Equation (25c)). In order to avoid the wastage of the energy, Equation (25d) is defined to restrict that the data packets must be transmitted within or at the maximum allowed gliding depth. In this way, the loss of data packets is avoided by keeping data packets within the gliding depth.

Let us suppose that the total available energy is $E_{total}$, which is computed by Equation (26).

(26) $$E_{total}=E_{tx}+E_{rx}$$

where $E_{tx}$ and $E_{rx}$ denote transmission energy and reception energy, respectively. These are calculated by the following Equation (27).

(27) $$E_{tx,rx}=P_{min,max(tx,rx)}\left(PacketSize/DataRate\right)$$

$P_{min,max(tx,rx)}$ shows the minimum and the maximum power required to transmit a data packet. It can be noted that the mechanism of allocating energy for receiving and transmitting a data packet gives the optimal results to the objective function.

We have used the graphical method because of two decision variables in our schemes, which are $E_{tx}$ and $E_{rx}$, and it is presented to analyze our objective function that the allocated energies are with in the feasible region.

**Figure 7:**
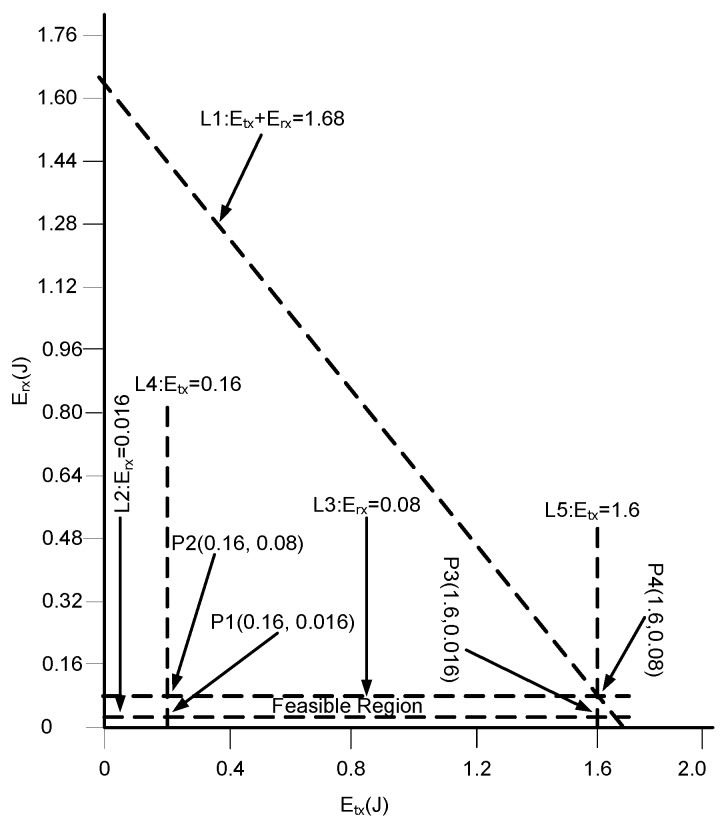
Feasible region for energy minimization.

Graphical analysis: Let us suppose that we have a scenario, in which the numbers of gliders are five to 45; $P_{tx}$ varies from 1 to 10 watt; $P_{rx}$ is from 0.1 to 0.5 watt; data rate = 10 Kbps; and packet size = 200 B. According to these values, from Equation (27), $E_{tx}$ is 1.6 joules when $P_{tx}$ is 10 watts and 0.16 at 1 watt. Similarly, $E_{rx}$ varies with the $P_{rx}$; it is 0.08 joules when $P_{rx}$ = 0.5 watt, and at 0.1 watt, it is 0.016 joules. Now, Equations (26) and (27) can written as (represented in joules):

at $P_{1}$: 0.16+0.016=0.176,
at $P_{2}$: 0.16+0.08=0.24,
at $P_{3}$: 1.6+0.016=1.616,
and at $P_{4}$: 1.6+0.08=1.68,

The intersection of lines L1, L2, L3, L4 and L5 is shown in Figure 7. The bounded region computed by these lines is the optimal region that is also called the feasible region; it satisfies all of the points and shows that a minimum energy will be consumed with the premise of that region resulting in a high network lifetime, leading to a comparatively better packet delivery ratio.

## 4. Performance Evaluation and Analysis

In order to evaluate our schemes (PAMP and Co PAMP), we compare these with the QUO VADISscheme. QUO VADIS has considered a predefined trajectory of gliders throughout the network lifetime. However, it makes the estimation process easy in internal uncertainty regions, but the coverage hole problem in sparse regions and the distance between neighbors during the estimation of external uncertainty regions are not considered, which results in low network throughput and high end to end delay. We have taken these factors into account, and the results are shown via simulations and discussed in this section.

Simulation parameters [1] of PAMP and Co PAMP are given in Table 4. The network volume is $2500\times 2500\times 1000\;m^{3}$; the number of gliders varies (5, 15, 25, 35, 45) in the network volume to avoid void holes. The transmission power also varies between 1 and 10 watt (W) with different operating frequencies of (10,15,25) kHz. The gliding depth range (R) is 0 to 1000 m with a speed (s) of 0.25 m/s, and the confidence parameter *β* is 0.05.

We use the following performance metrics for evaluating our proposed schemes.

- Packet delivery ratio: the number of correctly received data packets over the number of data packets sent.
- Energy consumption: the average amount of energy consumed to route one bit of information from the source to the destination. The unit of energy consumption is joule (J).
- Delay: the time taken by a data packet to reach the destination from the source. Its unit is second (s).
- Void zone: the network region that remains un-sensed or un-visited throughout the network lifetime. It is the ratio of the un-sensed region over the total volume of the network.

**Figure 8:**
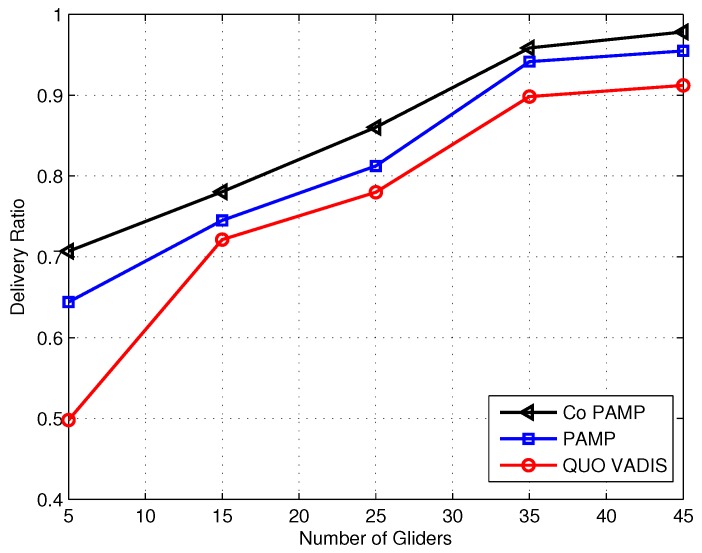
Delivery ratio comparison.

Figure 8 shows the delivery ratio of our proposed schemes and the existing compared scheme QUO VADIS. QUO VADIS follows a sawtooth trajectory and waits for the best network topology before the packet transmission. Due to the mobility pattern in QUO VADIS and the harsh underwater environment, the position and external-uncertainty region of the gliders cannot be estimated precisely. This uncertainty in estimation leads to packet drop and results in a low delivery ratio. PAMP uses the Markov process to predict sojourn positions of gliders, which decreases the error probability in the estimation of the position, resulting in a high delivery ratio. Compared to PAMP and QUO VADIS, Co PAMP has improved performance in terms of delivery ratio, as it transmits the packet in a cooperative manner. In Co PAMP, two best gliders are selected as relays from the set of neighboring gliders of the source glider, and after the packet transmission, SNR is calculated at the destination. If the SNR is low from the packet acceptance threshold, the first relay retransmits the packet received from the source glider, and again, SNR is computed. The process is repeated for the second relay, as well, if the SNR criteria are not met. As can be seen, as the number of gliders increases, the packet delivery ratio maximizes, as well. When numbers of gliders in Co PAMP are 5, 15 and 25, then the delivery ratio is 0.71, 0.78 and 0.85, respectively; whereas, QUO VADIS has a relatively lower packet delivery ratio as compared to Co PAMP. The acceptance in Co PAMP is high, due to the acceptance threshold, which is verified at each hop, and the gliders’ depth range is adjusted with respect to the link quality, which is computed using Equation (14). It is computed because with good link quality, the SNR of the received signal is high, leading to a high delivery ratio, as shown in the Figure 8.

**Figure 9:**
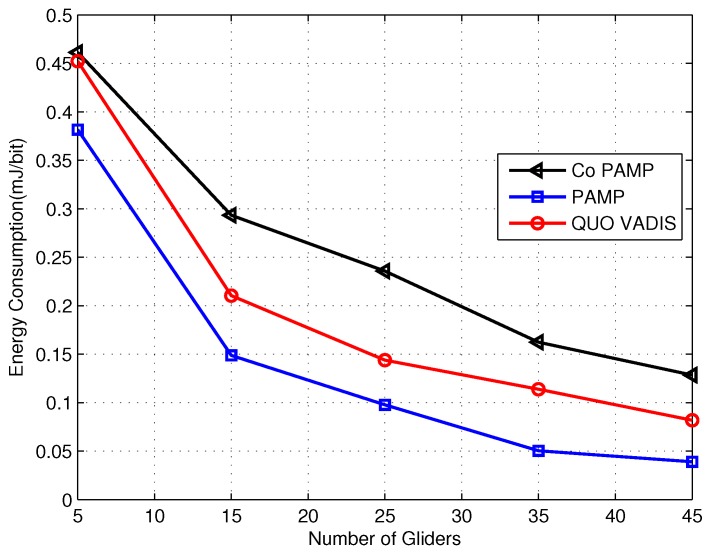
Energy consumption comparison.

The energy consumption comparison of the proposed schemes and QUO VADIS is shown in Figure 9. In QUO VADIS, gliders continuously move on a predefined trajectory, which makes the estimation of the external uncertainty region very difficult, which results in a high packet drop rate and high energy consumption. In PAMP, sojourn positions are introduced, and the continuous mobility is avoided due to which, uncertainty in position estimation is reduced; and the distance between the sending and the receiving glider remains constant for a specific time. On the other hand, the energy consumption of Co PAMP is higher than that of PAMP and QUO VADIS, as packets are retransmitted to achieve a high delivery ratio.

**Figure 10:**
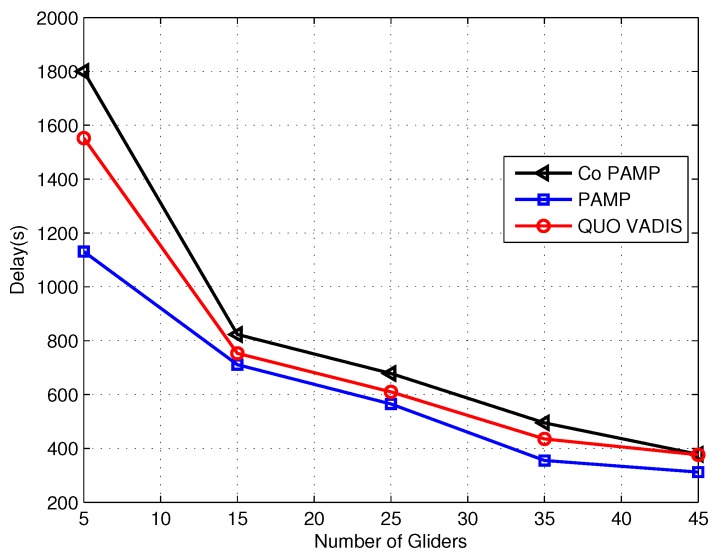
End-to-end delay comparison.

The delay comparison of QUO VADIS, PAMP and Co PAMP is shown in Figure 10. QUO VADIS waits for the best network topology to start communication, so its end to end delay is high. The end to end delay of PAMP is less than QUO VADIS and Co PAMP due to not waiting for the best network topology and the absence of cooperation, respectively. PAMP achieves high network throughput because gliders stop at sojourn positions to gather data, resulting in high end to end delay. In Co PAMP, high network throughput is achieved due to the cooperation of the two best relays gliders at the cost of high end to end delay. These relays retransmit the data of the source to the destination if SNR at the destination is not acceptable.

**Figure 11:**
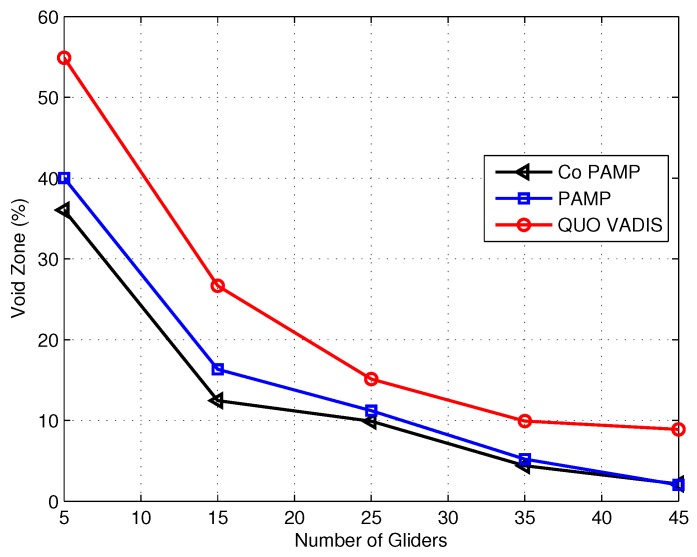
Void zone comparison.

The void zone or coverage hole is the volume of the network that remains unvisited (un-sensed) by network nodes or gliders. Figure 11 shows that QUO VADIS has more void zones compared to the proposed schemes due to the predefined mobility pattern (sawtooth trajectory). In the compared existing scheme (QUO VADIS), gliders do not change their course until the recharging process takes place, resulting in a high packet drop rate; whereas, in PAMP and Co PAMP, there is no such predefine mobility pattern; however, gliders move from dense to sparse network conditions to avoid void zones. In the case of five gliders, approximately 40% of the network volume of both proposed schemes is the void zone. As the number of gliders increased, we have seen a notable decrease in the void zone. In the proposed schemes, the void zone is avoided as two or more gliders are restricted not to follow the same course; whereas in Co PAMP, the void zone is almost similar to that of PAMP. The adjustment in the coordinates with the value of error (p) reduces the uncertainty and minimizes the void zones with the increase of the number of gliders. Figure 11 shows that when the number of gliders is 15, the percentage of void zones in Co PAMP is only 12%, whereas in the baseline scheme (QUO VADIS), the 28% network field is un-sensed. When the number of gliders is 45, then QUO VADIS has 9% of the field as void, and in our recent proposed scheme, Co PAMP has only a 3% volume of the network field as void. These stats show that the periodic mobility of gliders ensures that a very small volume of the network remains un-sensed in the recent proposed schemes (PAMP and Co PAMP).

### Performance Trade-Offs

Performance trade-offs of our proposed schemes and QUO VADIS are shown in Table 5. Gliders in QUO VADIS follow the sawtooth trajectory and wait for the best network topology, then they start communication. Future position and two uncertainty regions of gliders are estimated, to achieve a high delivery ratio, however at the cost of high end to end delay. In PAMP, gliders move randomly in the network area and periodically stay at sojourn positions. Transmission and reception of data are carried out at sojourn positions. PAMP achieves a high delivery ratio and less energy consumption as compared to QUO VADIS; however, the cost paid is high end to end delay. Co PAMP achieves a high delivery ratio as compared to both QUO VADIS and PAMP. In Co PAMP, cooperation is performed in which the two best neighboring gliders of the source act as relays and cooperate with the source glider to successfully deliver the data packet. The high delivery ratio in Co PAMP is achieved at the cost of high energy consumption and high end to end delay.

## 5. Conclusions and Future Work

We have proposed two routing protocols, PAMP and Co PAMP, to avoid void zones and achieve high network throughput. Due to the dynamic mobility pattern of gliders, void zones are avoided and energy is wasted due to the high packet drop ratio being minimized. For minimizing the uncertainty, predictable trajectories are used, and gliders stayed at static positions for a short interval to collect the data. The transmission power is adjusted according to the n-confidence region (neighboring nodes) for balanced energy consumption in estimating the s-confidence regions. In the estimation process of n-confidence and s-confidence regions, the distance between the source and the destination is considered, which helped in estimating the more precise position of the glider. The simulation results show that the recent proposed schemes (PAMP and Co PAMP) performed better than the baseline scheme (QUO VADIS) in terms of packet delivery ratio, energy consumption and void zone in the network.

In the future, we have planned to analyze the performance of gliders at different depths of the aquatic environment and the effects of interference at different depths due to noises. 

## Figures and Tables

**Table 1:** Performance tradeoffs in existing position estimation-based routing protocols.

Technique	Features	Achievements	Limitations
QUO VADIS [1]	Optimization technique based on position estimation and uncertainty and predefined trajectory of gliders	Low end to end delay	Transmission power is increased
Close-range tracking of AUVs [8]	Use of light beacon messages and camera to estimate the position of AUV	Exact and approximate position of the AUV is computed	Range is very short
SINS [9]	Positioning algorithm based on the strap down inertial navigation system (SINS) and the long baseline (LBL) positioning system	Exact position of the AUV is estimated	High energy consumption
AUV state estimation [10]	Kalman filter is used to estimate the state of the AUV	Navigation and state estimation of the AUV is done	High energy consumption
Trajectory-aware routing [11]	Model for the position uncertainty of the underwater glider	Minimization in uncertainty or error	High packet drop ratio

**Table 2:** Performance tradeoffs in existing mobility-based routing protocols. AURP, AUV-aided underwater routing protocol; SM, sink mobility; AEDG, an efficient data-gathering; CARP, channel-aware routing protocol.

Technique	Features	Achievements	Limitations
AURP [12]	Long data transmissions are minimized using AUVs as relay nodes	High delivery ratio and low energy consumption	High end to end delay
3D-SM [13]	Use of MS and courier nodes to transmit data	Energy consumption of normal nodes is minimized	High end to end delay
Distributed data gathering [14]	Routing using clusters and collection of data from path nodes using AUVs	Adjustment in transmission power	Overall transmission power is not minimized
AEDG [15]	Association of sensor nodes with gateway nodes	Reliability of data delivery at the destination	High end to end delay
EBRP (Energy Balanced Routing Protocol) [16]	Depth, residual energy and density of sensor nodes are considered as routing metrics	Energy of sensor nodes is saved and consumed in a balanced way	Low network lifetime
LAFR [17]	Link-state-based adaptive feedback routing and link state information is used for adaptive feedback	Utilization of asymmetric links and routing tables is maintained	Low network lifetime
CARP [18]	Selection of relay nodes that exhibit the latest history of successful data transmission	Loops are avoided, and data are routed around void and shadow zones	High end to end delay and more energy consumption
VAPR [19]	Void-aware pressure routing and sonobuoy propagates the control information	Avoidance of void zone	High end to end delay
Coverage hole avoidance [20]	Repairing of the coverage hole during network operation	Low energy consumption, high throughput and network lifetime	High end to end delay

**Table 3:** Performance tradeoffs in existing cooperation-based routing protocols. UASN, underwater acoustic sensor network; MCCR, minimum collision cooperative routing.

Technique	Features	Achievements	Limitations
Pressure routing for UASNs [21]	Cooperative routing and relay nodes are selected that are facing towards the destination	High throughput	High energy consumption
Depth and Energy Aware Dominating Set [22]	Single and multiple relay cooperative routing	High throughput and low packet drop ratio	High energy consumption
MCCR [23]	Cross-layer cooperative routing	Minimized collision	High end to end delay
Cooperative transmission [24]	Cooperative routing in a multi-hop network	Increased the packet delivery ratio and reduced the end-to-end delay	Nodes with reliable link die quickly
Optimal schemes [26]	Formulated problem with MINLP and solved using branch and bound algorithm	Reduced search space of the algorithm	The mechanism is not applicable to a dynamic topology
Co-UWSN [27]	Destination and relay node are selected using cost function, which depends on distance and SNR of the link	Considerable low end to end delay and less energy consumption	Redundant data forwarding and ACK mechanism on every packet is costly

**Table 4:** Simulation parameters of the proposed schemes.

Parameter	Values
Network volume	2500×2500×1000 m${}^{3}$
Number of gliders	[5, 15, 25, 35, 45]
Confidence parameter *β*	0.05
Power (min, max)	(1–10) W
Velocity of glider (s)	0.25 m/s
Gliding depth range (R)	[0–1000] m
error (p)	2 m
Operating frequencies (f)	[10, 15, 25] kHz

**Table 5:** Performance trade-offs in the proposed and compared schemes.

Protocol	Achieved Parameters	Figure	Compromised Parameter	Figure
QUO VADIS	Delivery ratio	Figure 8	Delay	Figure 10
PAMP	Delivery ratio energy consumption	Figure 8	Delay	Figure 10
		Figure 9		
Co PAMP	Delivery ratio	Figure 8	Energy consumption delay	Figure 9
				Figure 10
